# Methamphetamine Inhibits the Glucose Uptake by Human Neurons and Astrocytes: Stabilization by Acetyl-L-Carnitine

**DOI:** 10.1371/journal.pone.0019258

**Published:** 2011-04-27

**Authors:** P. M. Abdul Muneer, Saleena Alikunju, Adam M. Szlachetka, James Haorah

**Affiliations:** Laboratory of Neurovascular Oxidative Injury, Department of Pharmacology and Experimental Neuroscience, University of Nebraska Medical Center, Omaha, Nebraska, United States of America; Biological Research Center of the Hungarian Academy of Sciences, Hungary

## Abstract

Methamphetamine (METH), an addictive psycho-stimulant drug exerts euphoric effects on users and abusers. It is also known to cause cognitive impairment and neurotoxicity. Here, we hypothesized that METH exposure impairs the glucose uptake and metabolism in human neurons and astrocytes. Deprivation of glucose is expected to cause neurotoxicity and neuronal degeneration due to depletion of energy. We found that METH exposure inhibited the glucose uptake by neurons and astrocytes, in which neurons were more sensitive to METH than astrocytes in primary culture. Adaptability of these cells to fatty acid oxidation as an alternative source of energy during glucose limitation appeared to regulate this differential sensitivity. Decrease in neuronal glucose uptake by METH was associated with reduction of glucose transporter protein-3 (GLUT3). Surprisingly, METH exposure showed biphasic effects on astrocytic glucose uptake, in which 20 µM increased the uptake while 200 µM inhibited glucose uptake. Dual effects of METH on glucose uptake were paralleled to changes in the expression of astrocytic glucose transporter protein-1 (GLUT1). The adaptive nature of astrocyte to mitochondrial β-oxidation of fatty acid appeared to contribute the survival of astrocytes during METH-induced glucose deprivation. This differential adaptive nature of neurons and astrocytes also governed the differential sensitivity to the toxicity of METH in these brain cells. The effect of acetyl-L-carnitine for enhanced production of ATP from fatty oxidation in glucose-free culture condition validated the adaptive nature of neurons and astrocytes. These findings suggest that deprivation of glucose-derived energy may contribute to neurotoxicity of METH abusers.

## Introduction

Methamphetamine (METH) is the second most popular illicit drug widely used in the world. It is very prevalent in Western, Southern and Midwestern states of USA [1]. The escalating problems due to METH abuse in these states cost enormous financial and health burdens to family and society. The adverse effects of METH abuse include addiction, impairment of behavioral and cognitive function, and neurotoxicity [2], [3], [4], [5]. METH abuse is known to promote neurotoxicity by altering dopamine levels [6], as such initial accumulation and long-term depletion of dopamine in the brain causing loss of dopaminergic neurons [7], [8]. Acute high doses of METH lead to hyperthermia and neurotoxicity with dopamine depletion, while chronic METH abuse seems to cause hypothermia without depletion of dopamine [9]. Interestingly, accumulation of dopamine in chronic self-administration of METH triggers the activation of microglia and loss of neurons in human brain [10], [11]. Exacerbated dopaminergic neuronal death was also demonstrated by dopamine overloading [12]. Initial dopamine accumulation and gradual long-term dopamine depletion associating with neurotoxicity is a typical mechanism of action of METH abuse [13], [14]. This is because the ability of METH to release dopamine rapidly and inhibits the reuptake, and/or perhaps blocking the metabolism of dopamine in the reward regions produces the euphoric feeling to METH abusers.

The induction of oxidative stress in dopaminergic neurons also supports the role of dopamine in METH mediated neurotoxicity [15]. Recently, Ramirez et al. (2009) and Sharma et al. (2010) demonstrated the METH-elicited disruption of BBB and neurotoxicity as a result of oxidative stress [16], [17]. These reports are in line with the findings that antioxidants attenuate METH-induced neuronal degeneration [18]. Interestingly, METH-induced neuronal degeneration is often associated with the activation of astroglial cells (astrocyte and microglia) in the brain [19], [20]. One common beneficial mediator for the survival of these cells is governed by glucose uptake and metabolism. Therefore, impairment of this glucose regulation by METH is expected to be detrimental to the survival of these neuro-glial cells (neuron, astrocyte and microglia). METH appeared to disrupt the metabolism of glucose in the frontal cortex [21], thalamus and striatum [22]**,** and limbic areas of the brain [23].

To date there is no record of studies that demonstrate the effects of METH on glucose uptake and glucose transporter in primary human neurons and astrocytes. In this study, we hypothesized that METH exposure may interfere with astrocytic glucose transporter protein-1 (GLUT1) and neuronal GLUT3 function. GLUT1 and GLUT3 are the principal glucose transporters that facilitate the transport of glucose in the brain [24], [25]. GLUT1 exists as 55 kDa and 45 kDa isoforms, of which the highly glycosylated 55 kDa GLUT1 isoform is localized exclusively in brain endothelial cells [26], [27]. The less glycosylated 45 kDa GLUT1 isoform is expressed in the perivascular end-feet of astrocytes [28] and the 45-60 kDa GLUT3 is localized exclusively in neurons [25]. Our findings revealed that human neuronal GLUT3 and astrocytic GLUT1 are affected by METH exposure. The use of GLUT inhibitor cytochalasin B validated the importance of glucose uptake and metabolism for the survival of these brain cells.

## Materials and Methods

### Reagents

Antibodies to GLUT1, GLUT3, glial fibrillary acidic protein (GFAP, astrocytes marker) and neurofilament (NF, neuronal marker) were purchased from Abcam (Cambridge, MA). Antibody to α-actin was from Millipore (Billerica, MA). All secondary Alexa Fluor antibodies were purchased from Invitrogen. D-(2-^3^H)-glucose (5 mCi, 185 MBq) was purchased from PerkinElmer Life and Analytical Sciences (Waltham, MA). Cytochalasin B, acetyl-L-carnitine (ALC, cofactor of β-oxidation) and 3-(4,5-Dimethylthiazol-2-yl)-2,5-diphenyl-tetrazolium bromide (MTT) were purchased from Sigma-Aldrich (St. Louis, MO).

### Cell culture

Cortical neurons and astrocytes were obtained from our neural tissue core facility. We routinely isolate these cells from elective abortus specimens of human fetal brain tissues in our core facility. Tissues were obtained in full compliance with the ethical guidelines of both the National Institutes of Health (NIH) and the University of Nebraska. Briefly, dissociated tissues were incubated with 0.25% trypsin for 30 min, neutralized with 10% fetal bovine serum, and further dissociated by trituration. Neurons were cultured on poly-D-lysine pre-coated cover slips and 6 well plates (BD Labware, Bedford, MA) in Neurobasal™ Medium containing 0.5 mM glutamine, 50 µg/ml each of penicillin and streptomycin in combination with GIBCO™B-27 supplements with antioxidants as described previously [29]. Astrocytes were cultured in DMEM/F-12 media containing HEPES (10 mM), sodium bicarbonate (13 mM, pH 7), 10% fetal bovine serum, penicillin and streptomycin (100 µg/ml each, invitrogen) as described [30]. Purity of neurons was assessed by MAP-2 antibody (Chemicon) and astrocytes by GFAP antibody, which normally showed 100% enrichment of neurons or astrocytes. For glucose uptake and cell viability assays, cells were cultured in 96-well plates (20,000 cells/well). Cells were plated on 12-well plates containing glass cover slips (40,000 cells/well) for immunocytochemistry. For protein extractions, astrocytes were cultured in T 75 cm^2^ flasks (1×10^6^ cells/flask) and neurons were cultured in 6 well plates (0.2×10^6^ cells/well). Cell culture media was changed every 3rd day until cells were confluent (4–5 days for astrocytes and 10–12 days for neurons).

### Cell viability assay

Cell viability was determined by 3-(4,5-dimethylthiazol-2yl)-2,5-diphenyl tetrazolium bromide (MTT) assay. The assay is based on the cleavage of yellow tetrazolium salt to purple formazan crystals by metabolically active cells. Briefly, cells cultured in 96-well microtiter plates were added 100 µl MTT (5 mg/ml MTT in 10% FBS in 1X PBS) after treatments with test compounds for appropriate time points. Cells were then incubated at 37°C for 45 minutes. Then 100 µl DMSO was added just after aspirating the MTT solution and the plates were incubated at room temperature for 15 min. Absorbance of the purple formazan was detected by a microtiter plate reader at 490 nm wavelength.

### Glucose uptake assay

Following the modified method of Takakura [31], D-(2-^3^H)-glucose uptake was performed on fully confluent human astrocytes and neurons cultured in 96 well plates. Cells were exposed to 20 µM and 200 µM METH (for astrocytes) and 20 µM and 100 µM METH (for neurons) for 24 hr in the presence or absence of 10 µM cytochalasin B (10 mM stock dissolved in DMSO) or 100 µM ALC. Cells were then incubated overnight in glucose-free DMEM/F-12 media (for astrocytes) and glucose-free neurobasal media (for neurons) containing equimolar of D-(2-^3^H)-glucose (1.0 µCi) and non-radiolabeled glucose. After washing off the excess ^3^H-glucose with Krebs-Ringer phosphate-HEPES (KRPH) buffer, cellular protein was precipitated with 10% TCA at 4°C for 15 min. Precipitated proteins were transferred onto a 96 well nitrocellulose filter using the Unifilter-96 well Harvester as per the manufacturer's instructions (PerkinElmer, Waltham, MA). Using a Beckman 96 well plate reader, radioactivity was measured by β-top counter.

### Immunocytochemistry

For immunocytochemistry, primary human astrocytes and neurons were cultured on glass cover slips in 12 well plates until 80–100% confluent. Cells were then treated with 20 µM (for neuron and astrocytes) and 200 µM of METH for astrocytes or 100 µM of METH for neurons in the presence or absence of cytochalasin B (10 µM) or ALC (100 µM) for 24 hours. Cells were washed with PBS and fixed in ice-cold acetone-methanol (1∶1 v/v). After blocking the cellular antigen with 3% bovine serum albumin at room temperature for 1 hr in the presence of 0.1% Triton X-100, cells were incubated overnight at 4°C with respective primary antibodies: mouse anti-GLUT1 (1∶250 dilution) and rabbit anti-GFAP (1∶200 dilution) for astrocytes; rabbit anti-GLUT3 (1∶250 dilution) and mouse anti-NF (1∶250 dilution) for neurons. Cells were washed and then incubated for 1 hr with secondary antibodies; anti-mouse-IgG Alexa Fluor 594 for GLUT1, anti-rabbit-IgG Alexa Fluor 488 for GFAP, anti-rabbit-IgG Alexa Fluor 594 for GLUT3 and anti-mouse-IgG Alexa Fluor 488 for NF. Cover slips were then mounted onto glass slides with immunomount containing DAPI (Invitrogen), and then fluorescence microphotographs were captured by fluorescent microscopy (Eclipse TE2000-U, Nikon microscope, Melville, NY) using NIS-Elements (Nikon, Melville, NY) software.

### Western blotting

Astrocytes cultured in T-75 cm^2^ flasks and neurons cultured in 6 well plates were lysed with CellLytic-M (Sigma) for 30 min at 4°C, centrifuged at 14000 x g, and cell lysates protein concentrations in the supernatants were estimated by BCA (Thermo Scientific, Rockford, IL). We loaded 20 µg protein/lane and resolved the proteins by SDS-PAGE on 4-15% gradient gels (Thermo Scientific) and then transferred the protein onto nitrocellulose membranes. After blocking with Superblock T-20 (Thermo Scientific, Rockford, IL) membranes were incubated for overnight with primary antibody against GLUT1 for astrocytes and GLUT3 for neurons (1∶1000, Abcam, Cambridge, MA) at 4°C followed by 1 hr incubation with secondary antibodies conjugated with horse-radish peroxidase. Immunoreactive bands were detected by West Pico chemiluminescence substrate (Thermo Scientific) using an autoradiography developer. Data were quantified as arbitrary densitometry intensity units by Gelpro32 software package (Version 3.1, Media Cybernetics, Marlow, UK).

### ATP production assay

Using pyruvate (PVA, 4.0 mM) and palmitate (PA, 4.0 mM) as substrates, the production of adenosine triphosphate (ATP) via mitochondrial β-oxidation was determined by ATP determination kit (Molecular Probes, Eugene, OR) in glucose-free neuronal and astrocytic cell cultures as described by Drew and Leeuwenburgh [32]. The standard curve was extrapolated from 0.125, 0.25, 0.5, 1.0, 2.0, 4.0, 8.0, and 16.0 µM concentrations of ATP. The reaction mixture was maintained at 28°C, and the luciferase assay for ATP production was performed on fluorescence plate reader with luminometer function (M5, Molecular Devices, Sunnyvale, CA) using 96 well plates. ATP levels were normalized to milligram cellular protein derived from the protein estimation of the 96 well plates by BCA method.

### Statistical analysis

All result values are expressed as the mean ± SEM. Within an individual experiment, each data point was determined from three to five replicates. Statistical analysis of the data was performed using GraphPad Prism V5 (Sorrento Valley, CA). Comparisons between samples were performed by one-way ANOVA with Dunnett's post-hoc test. Differences were considered significant at P values ≤0.05.

## Results

### Neurotoxicity of METH and its effect in glucose utilization

**Figure 1:**
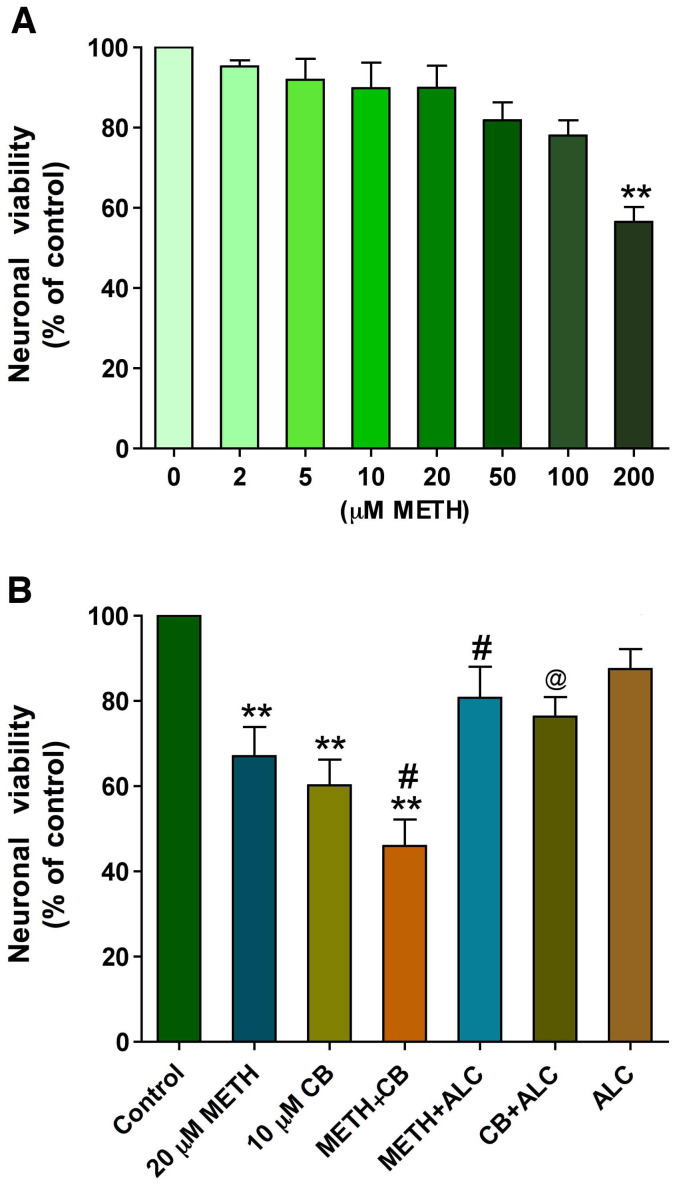
Effect of METH-induced glucose deprivation on neuronal viability: **A.** Dose dependent effects of METH on cell viability in human neurons at 24 hr. **B.** Cell viability assay of human neurons treated with 20 µM METH in presence or absence of cytochalasin B (10 µM) or ALC (100 µM) for 72 hr. Statistically significant (n = 5), **p<0.01 compared with controls in **A** and **B**; ^#^P<0.05 with 20 µM METH in **B**; and ^@^p<0.05 with 10 µM cytochalasin B in **B**.

We first determined the dose-dependent effects of METH (2 - 200 µM) on neuronal toxicity. Our data indicate that treatment of neurons with METH concentrations of 2 - 100 µM for 24 hr had no significant effect on cell viability, but the high concentration (200 µM) of METH affected neuronal survival, which was attributed to direct toxicity of METH (Fig. 1A). In order to evaluate whether the long-term exposure of low METH concentration (20 µM) can affect neuronal survival by inhibiting glucose uptake, we analyzed the viability of neurons in the presence or absence of GLUT inhibitor cytochalasin B at non-toxic level of 10 µM. Concentration of cytochalasin B higher than 20 µM almost completely inhibited GLUT3 function in primary human neuronal culture causing neurotoxicity (data not shown). The non-toxic concentration of cytochalasin B (10 µM) was derived from dose- and time-dependent toxicity assay using cytochalasin B concentrations of 0.5–100 µM treatment for 24–72 hrs (data not shown). Optimization of non-toxic concentration was important because cytochalasin B has diverse effects on cellular function including cell division, actin polymerization and platelet aggregation [33]. Neurons cultured in 96 well plates were treated for 72 hrs and analyzed for cell viability by MTT assay. The results showed that although 20 µM METH and 10 µM cytochalasin B individually affected cell survival, combination of METH and cytochalasin B exacerbated the loss of cell viability (Fig 1B). Since cytochalasin B exacerbated the effect of METH, these data suggest that the impairment of glucose transporter function may contribute to neuronal loss in chronic METH exposure. The protective effect of ALC from METH and cytochalasin B in part supported the notion that an alternative energy utilization pathway or oxidative stress may be involved for neuronal loss during intracellular glucose deprivation.

### METH exposure inhibits GLUT3 and glucose uptake in neurons

**Figure 2:**
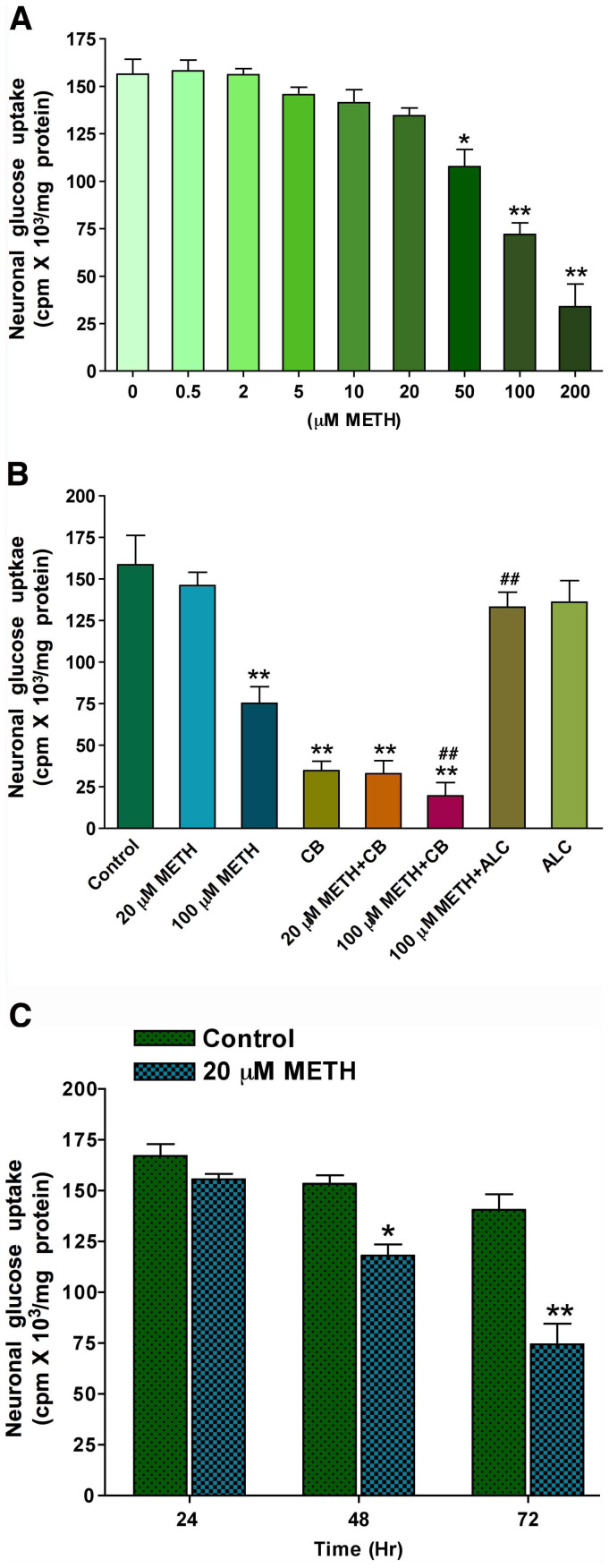
METH down regulates glucose uptake in human neurons: **A.** Dose dependent effects of METH on D-(2-^3^H)-glucose uptake by human neurons for 24 hr. **B.** Effects of GLUT inhibitor cytochalasin B (10 µM) and ALC (100 µM) on glucose uptake by neurons following 20 µM and 100 µM of METH for 24 hr. **C.** Effect of 20 µM METH on D-(2-^3^H)-glucose uptake in human neurons at 24, 48 and 72 hr exposure periods and compared with respective untreated control cells. Statistically significant, *p<0.05, **p<0.01 compared with controls (n = 5 in **A** and **B**, n = 3 in **C**); ^##^p<0.01 with 100 µM of METH (in **B**, n = 5).

To evaluate whether METH exposure can disrupt glucose uptake, we treated neuronal culture with 20 µM and 100 µM of METH for 24 hr. Cells were then used for glucose uptake assay and extraction of protein for analysis of GLUT3 protein levels. The uptake of glucose by neurons decreased gradually with increasing METH concentrations dose-dependently (Fig 2A). We tested the inhibitory (with cytochalasin B) and protective (with ALC) effects on neuronal glucose uptake using low and high concentrations of 20 µM and 100 µM of METH. As expected, cytochalasin B inhibited the rate of glucose uptake in presence or absence of METH, however ALC effectively protected neurons from the adverse effect of 100 µM METH on glucose uptake (Fig 2B). Note that the presence of cytochalasin B exacerbated the inhibitory effects of METH on glucose uptake at both tested concentrations, suggesting that interference of neuronal glucose transport and metabolism by METH may contribute to neuronal degeneration.

**Figure 3:**
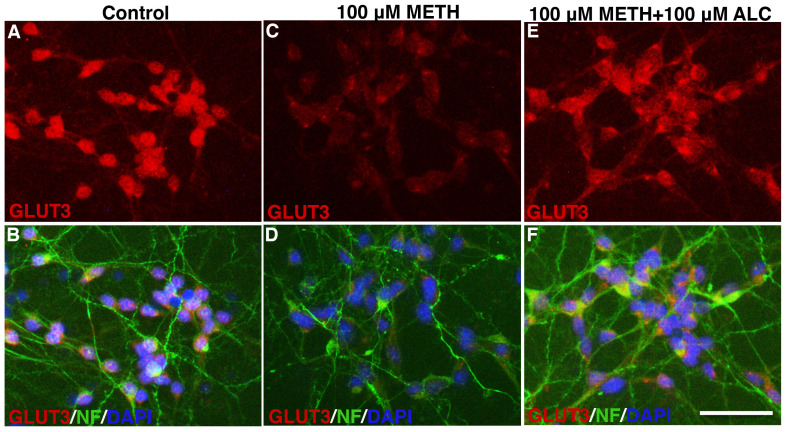
Effects of METH on GLUT3 expression in human neurons: Immunocytochemistry of GLUT3 (red) merged with NF (green) and DAPI (blue) in control (**A–B**), 20 µM METH (**C–D**), and 100 µM METH+100 µM ALC (**E–F**). Rabbit anti-GLUT3 and mouse anti-NF were used as primary antibodies for overnight at 4°C. Anti-rabbit-IgG Alexa Fluor 594 for GLUT3 and anti-mouse-IgG Alexa Fluor 488 for NF were used as secondary antibodies. Scale bar indicates 10 µm in all panels.

**Figure 4:**
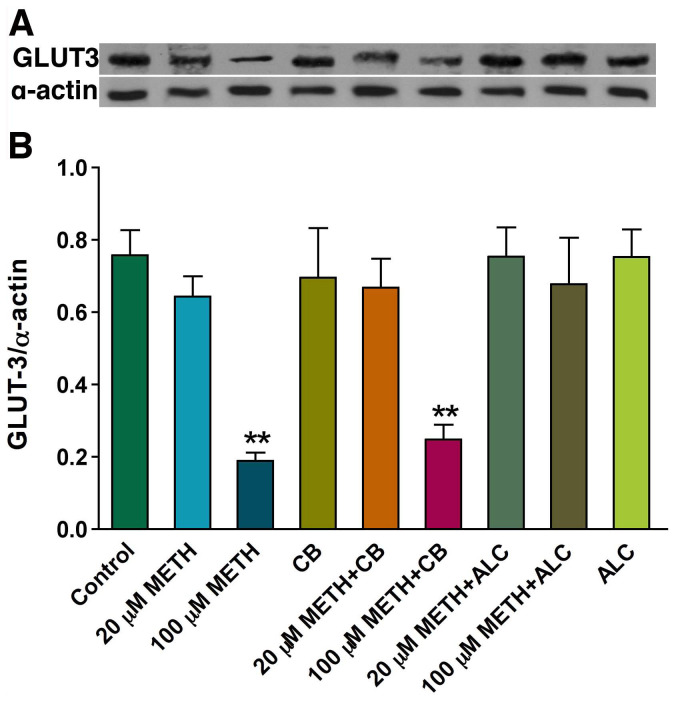
Western blot analysis for GLUT3 expression in METH treated human neurons. **A-B:** Effects of METH on GLUT3 (45 kDa) protein levels in human neurons. Bar graph shows the results, which are expressed as ratio of GLUT3 to that of α-actin bands, and presented as the mean values (± SEM; n = 5). (Statistically significant, **p<0.01 compared with controls).

In agreement with reduction in glucose uptake, we found that METH exposure highly reduced the expression of GLUT3 protein in primary human neuronal cultures (Fig 3A–F). Our results indicate that primary human neurons expressed only GLUT3 (not GLUT1, data not shown), and expression of GLUT3 exclusively in neurons was reported by Simpson et al. 2008 [25]. We observed that GLUT3 was localized mostly in neuronal cell body and not in neurofilaments (Fig 3A–F). We also validated the effects of cytochalasin B, ALC and METH on GLUT3 protein levels by Western blot analyses. Our data demonstrated a significant diminution of 45 kDa GLUT3 protein levels by 100 µM METH exposure and protective effect by ALC compared with the control (Fig 4A–B). Treatment of neurons with cytochalasin B did not alter GLUT3 protein levels (Fig 4A–B), suggesting that cytochalasin B inhibits glucose uptake by blocking the active binding of GLUT3 in neurons, but not by reducing the level of GLUT3 protein.

### Effects of METH on survival of astrocytes

**Figure 5:**
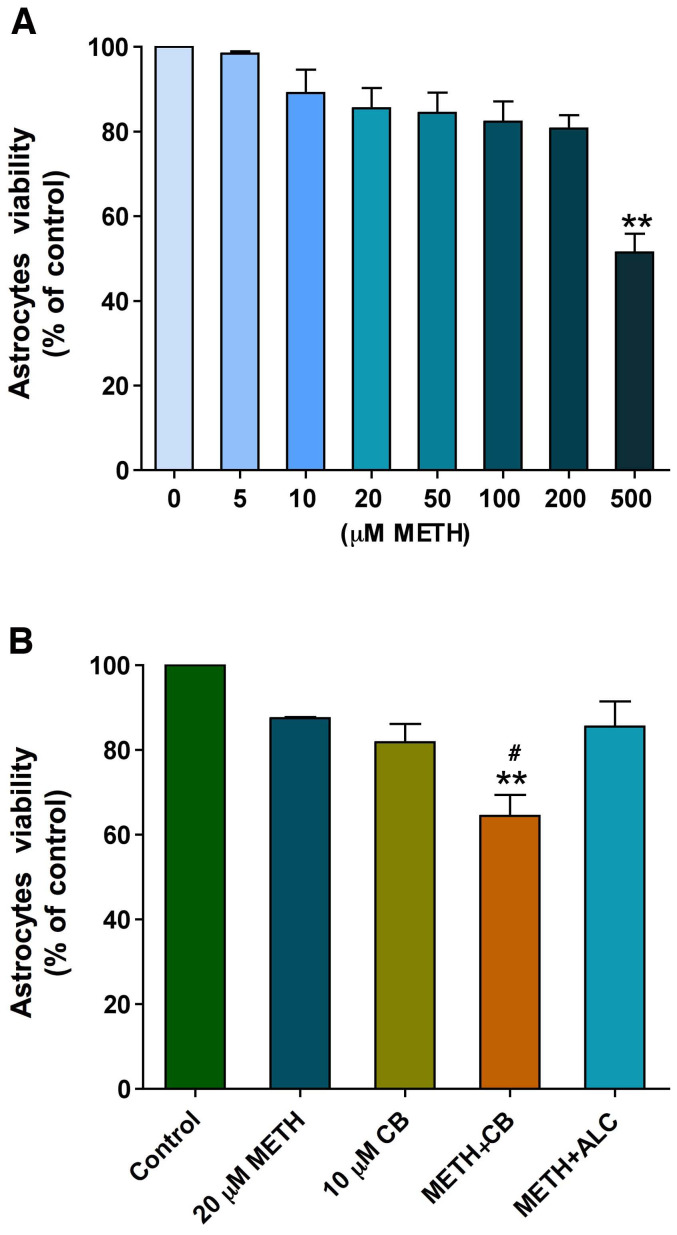
METH-induced glucose deprivation cause cell death in human astrocytes: **A.** Dose dependent effects of METH on cell viability in human at 24 hr. **B.** Cell viability assay of human astrocytes treated with 20 µM METH in presence or absence of cytochalasin B (10 µM) or ALC (100 µM) for 72 hr and compared with untreated cells. Statistically significant (n = 5), **p<0.01 compared with controls in **A** and **B** and ^#^p<0.05 with 20 µM METH or 10 µM cytochalasin B in **B**.

Next, we tested the dose-dependent effects of METH on viability of primary human astrocytes in culture. METH concentrations of 5**–**200 µM had no significant effect on cell toxicity while 500 µM METH showed significant toxic effect to astrocytes (about 50% cell death compared with control) following 24 hr exposure (Fig 5A). We also evaluated the survival of astrocytes after long-term exposure of non-toxic METH concentration (20 µM). The use of non-toxic level cytochalasin B (10 µM, GLUT inhibitor) was to examine the possible role of glucose mishandling as contributing factor for cellular death in chronic METH exposure at physiologically detected level. For this reason, astrocytes cultured in 96 well plates were treated with METH for 72 hrs and analyzed for cell viability by MTT assay. Although there was a pattern of reduction, our results showed that 10 µM cytochalasin B or 20 µM METH individual treatment had no significant effect on cell viability unlike the combination of cytochalasin B and METH (Fig 5B). These results suggest that long-term exposure of low dose METH may disrupt glucose regulation and survival of astrocytes.

### Effects of METH on human astrocytic glucose uptake

**Figure 6:**
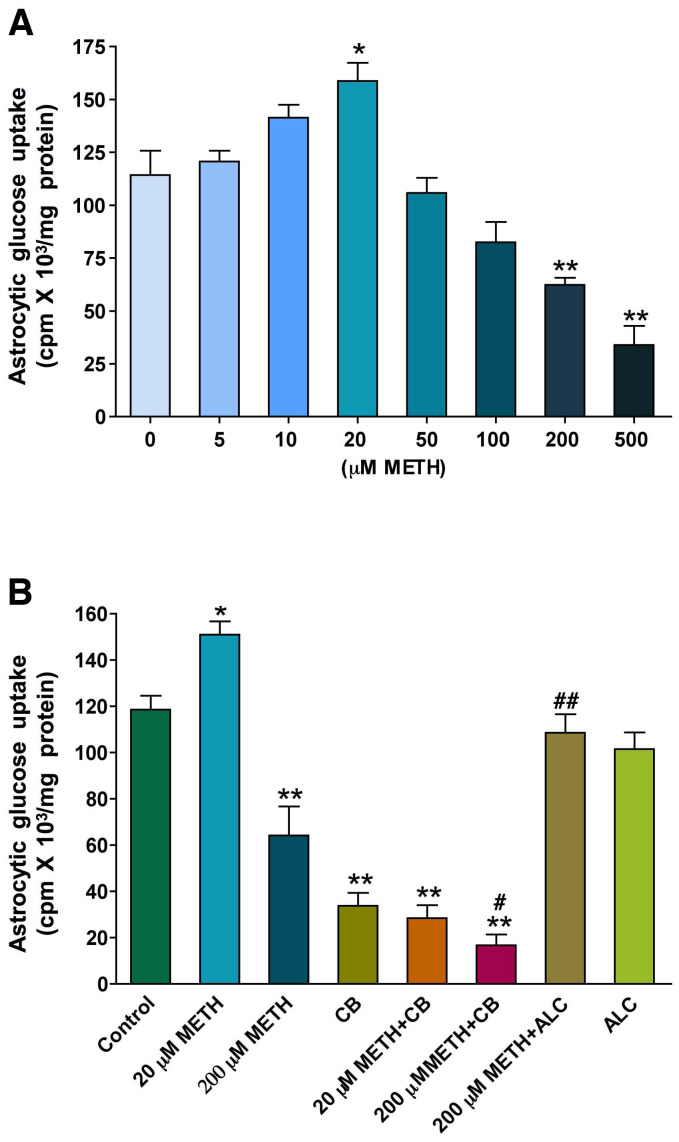
Dual effects of METH on glucose uptake in astrocytes: **A.** Dose dependent effects of METH on D-(2-^3^H)-glucose uptake by human astrocytes for 24 hr. **B.** Effects of GLUT inhibitor cytochalasin B (10 µM) and ALC (100 µM) on glucose uptake by astrocytes following 20 µM and 200 µM of METH for 24 hr treatment. Statistically significant (n = 5), *p<0.05, **p<0.01 compared with controls in **A** and **B** and ^#^P<0.05, ^##^p<0.01 with 200 µM METH in B (third bar).

To correlate the loss of astrocytes with inhibition of glucose uptake by these cells, we studied the dose-dependent effects of 5 – 500 µM METH on glucose uptake after exposing for 24 hr in culture media. Surprisingly, we found that 5 – 20 µM METH increased glucose uptake dose-dependently and above 20 µM METH (50**–**500 µM) decreased glucose uptake gradually (Fig 6A). Since there was a marked differential effect between 20 µM and 200 µM of METH on astrocytic glucose uptake, we studied the inhibitory effect of cytochalasin B (10 µM) and the protective effect of ALC (100 µM) on glucose uptake with these two METH concentrations. The ALC concentration of 100 µM was derived from dose dependent study of 50**–**500 µM on cell viability (data not shown). Interestingly, ALC stabilized the inhibitory effect of 200 µM METH on glucose uptake to control level, suggesting that ALC may ameliorate the impairment of glucose uptake (Fig 6B). As expected, cytochalasin B inhibited the effect of 20 µM and exacerbated the effect of 200 µM METH on glucose uptake, suggesting the direct involvement of GLUT1 in human astrocytes (Fig 6B).

### Effects of METH on astrocytic GLUT1 protein expression

**Figure 7:**
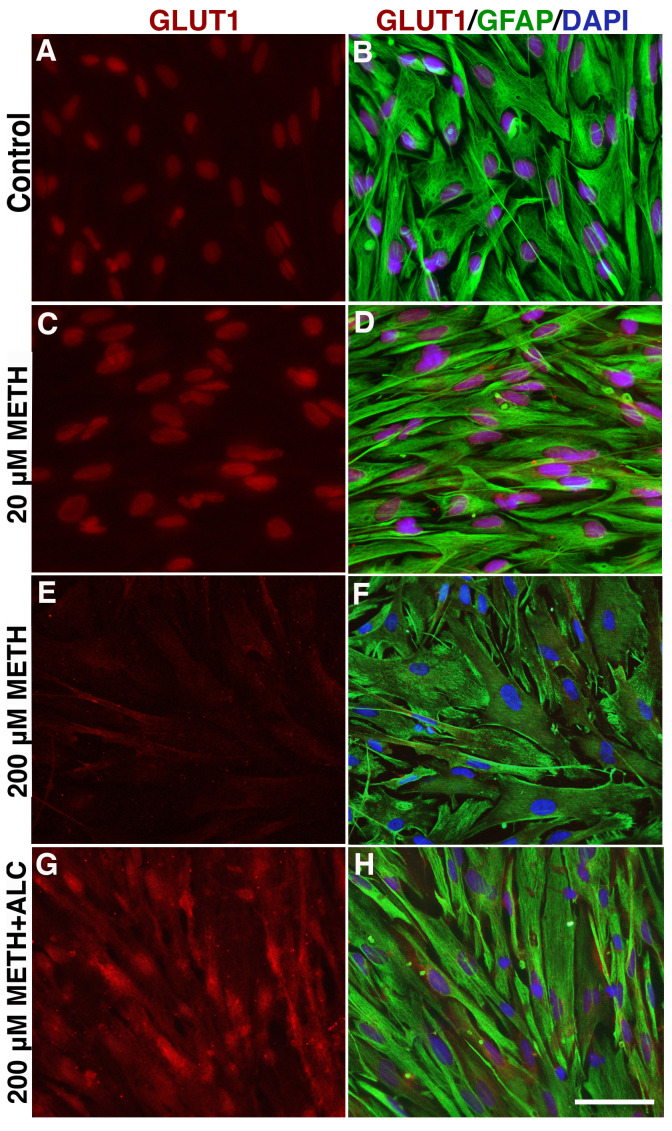
Expression of GLUT1 in METH treated human astrocytes: A-H: Immunocytochemistry of GLUT1 (red) merged with GFAP (green) and DAPI (blue) in human astrocytes. The expression of GLUT1 is shown in control (**A–B**), 20 µM METH (**C–D**), 200 µM METH (**E–F**) and 200 µM METH+100 µM ALC (**G–H**). Mouse anti-GLUT1 and rabbit anti-GFAP were used as primary antibodies for overnight at 4°C. Anti-mouse-IgG Alexa Fluor 594 for GLUT1 and anti-rabbit-IgG Alexa Fluor 488 for GFAP were used as the secondary antibodies. Scale bar indicates 10 µm in all panels.

**Figure 8:**
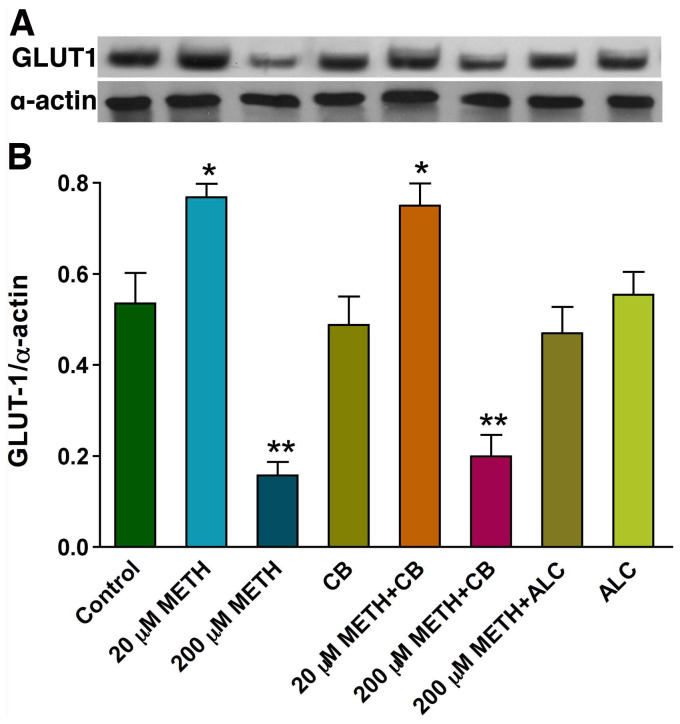
Western blot analysis of GLUT1 in METH treated human astrocytes: **A:** Effects of METH on 45 kDa GLUT1 protein levels in human astrocytes. The effects of cytochalasin B and ALC on GLUT1 expression also showed in the band pattern. **B.** Results are expressed as ratio of GLUT1 to that of α-actin bands, and presented as the mean values (± SEM; n = 5). (Statistically significant, *p<0.05 **p<0.01 compared with controls).

We then confirmed the expression of GLUT1 by immunocytochemistry. In agreement with glucose uptake data, low dose of METH (20 µM) increased the expression of GLUT1 protein, whereas 200 µM METH decreased the expression of GLUT1 (Fig 7A**–**F). ALC almost completely prevented the down regulation of GLUT1 protein expression by high dose METH (200 µM) in astrocytes (Fig 7E**–**H). In agreement with these findings, Western blot analyses validated the protective effect of ALC on GLUT1 protein content from 200 µM METH (Fig 8A**–**B). Unlike GLUT1 in brain microvessels that express 55 kDa isoform, we detected a distinct 45 kDa isoform GLUT1 protein in astrocytes (Fig 8A). We also noted that unlike the glucose uptake findings, cytochalasin B neither decreased the GLUT1 protein level from low dose METH nor exacerbated the effect of high dose METH on 45 kDa isoform GLUT1 protein levels in astrocytes (Fig 8A-B).

### Can neurons and astrocytes adapt to alternative energy metabolic pathway during glucose deprivation?

**Figure 9:**
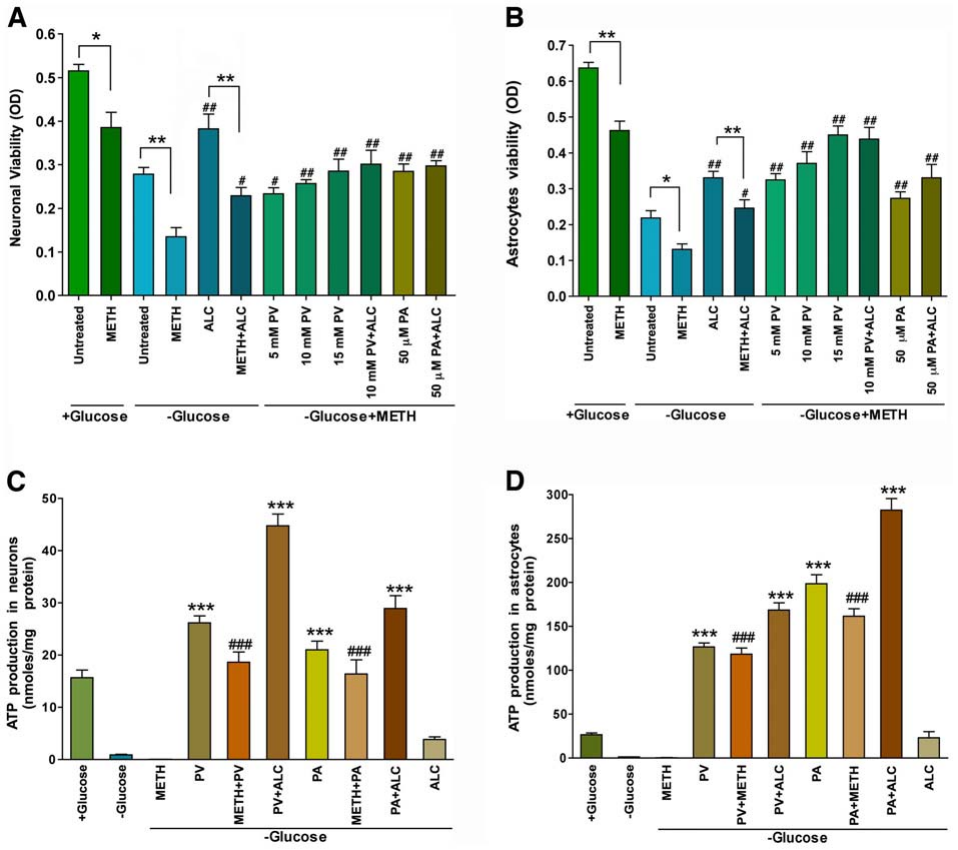
Utilization of pyruvate/palmitate by neurons and astrocytes during glucose deprivation and METH insult: Primary human neurons and astrocytes cultured in 96 well plates (20000 cells/well) in normal (+ glucose) or glucose-free (- glucose) media were exposed to 100 µM METH for 48 hr, and were analyzed for cell viability and ATP production. Viability of cells in glucose-deprived culture supplemented with pyruvate (PV)/palmitate (PA) in the presence or absence of ALC (100 µM) in (**A**) neurons and (**B**) astrocyte. Production of ATP in glucose-deprived culture with PV/PA in the presence or absence of ALC, (**C**) in neurons and (**D**) in astrocytes. Untreated (+) glucose is a positive control and untreated (-) glucose is a negative control. Results are expressed as mean values (± SEM; n = 3 in **A** and **B**; n = 5 in **C** and **D**). Statistically significant *p<0.05 and **p<0.01 compared with respective conditions (adjacent bars) in **A** and **B**; ***p<0.001 with compared with (-) glucose in **C** and **D**; ^#^p<0.05 and ^##^p<0.01 compared with (-) glucose+METH in **A** and B, and ^###^p<0.001 with compared with (-) glucose+METH condition in **C** and **D**.

We asked this question because blockade of glucose uptake by cytochalasin B (see Fig 6B) did not significantly affect astrocytic cell death (see Fig 5B), suggesting that astrocytes might have turned on to alternative energy metabolic mechanism. Also, because ALC was protective (ALC a key cofactor of β-oxidation), we tested the idea that these brain cells may be utilizing non-carbohydrate substrates as the source of ATP for survival during METH-induced mishandling of glucose. Thus, we assayed the mitochondrial oxidation of pyruvic acid (PV) and palmitic acid (PA) in neurons and astrocytic culture in glucose-free media for analyses of cell survival rate and ATP production. Our results indicate that reduction of neuronal and astrocytic viability in glucose deprivation was abrogated by 100 µM METH, which was mitigated by supplementation of pyruvate (dose-dependently) and palmitate at lower concentration in these two cell types (Figs 9A and B). Since 15 mM of pyruvate was already a high physiological concentration, we did not test the effect of PV above this level. We tested the effect of PA up to 2.0 mM and found that lower concentrations of 50**–**250 µM afforded better protection than the higher concentrations. PA concentration higher 2.0 mM was less soluble in media containing 0.02% DMSO.

Production of ATP from PV/PA was assessed to support the argument that survival of these cells in glucose deprivation was due to oxidation of PV/PA. In agreement with cell viability data, we found that both neurons and astrocytes were capable of oxidizing PV and PA in the presence or absence of METH, in which ALC significantly enhanced the levels of ATP (Fig 9C and D). However, it was apparent that astrocytes were better adaptive than neurons for oxidation of fatty acids as source of energy. These results suggest that brain cells like astrocytes and neurons do utilize alternative energy metabolic mechanism during METH-induced impairment of glucose utilization. These results suggest that METH appears to impair GLUT1 function and glycolysis without affecting Krebs cycle step that converts PV or PA to ATP as bio-energy.

## Discussion

We demonstrate here for the first time that METH exposure had deleterious effects on glucose uptake and glucose transporter proteins in primary human neurons and astrocytes culture. Our data suggest that deprivation of glucose and inability of brain cells to process the glucose for bio-fuel production is an important key contributing mechanisms for neuronal degeneration in METH abusers. Further, therapeutic application of ALC could be beneficial for improving the health of METH abusers.

Glucose is the main energy source for the brain, which is transported from the circulation to the brain by BBB endothelial GLUT1. GLUT1 then delivers glucose to astrocytes via the astrocytic 45 kDa GLUT1 isoform and to neurons via the neuronal GLUT3 protein. The glycolytic product of glucose such as pyruvate is transported into the neurons by endothelial and astrocytic monocarboxylate transporter1 (MCT1) and out of the neurons by neuronal MCT2 [24]. Interference in any of this shuttling process is expected to disrupt the regulation of glucose metabolism and energy utilization in the brain.

Therefore, inhibition of glucose uptake by methamphetamine (METH) that we observed in astrocytes and neurons indicate that glucose deprivation may be one of the putative mechanisms for neurotoxicity in METH abusers. The rationale for implicating these findings to pathophysiologic neurotoxicity in METH abusers evolved from the fact that these cells are primary human brain cells. Unlike astrocytes, neurons were very sensitive to METH exposure. One reason for the differential effects could be attributed to fetal origin and the adaptive nature of these cell types. Fetal neurons are very sensitive to exogenous stress agents, whereas glial cells like astrocytes are better adaptive to environmental stress. It remains open for further investigation whether neurons and astrocytes derived from adult brain will be less sensitive to METH insults. This adaptive nature may also explain the dual effects of METH on astrocytic glucose uptake. In that, the effect of 20 µM METH for increasing glucose uptake appeared to be an acute adaptive response of immune cells like astrocytes because we observed that chronic exposure of 20 µM METH decreased the glucose uptake in astrocytes. The low (20 µM) and high (200 µM) concentrations of METH that we used here were similar to the levels of METH detected in blood samples of recreational users [34], [35] and in chronic METH abusers [36].

It was also observed that astrocytes had better adaptive response to utilization of non-carbohydrate substrate as an alternative energy source during glucose-deprived stress condition. Such was the case here when glucose delivery was limited by METH exposure, ALC protected neurons and astrocytes from the adverse effects of METH, suggesting the activation of fatty acid oxidation. ALC is the primary cofactor of mitochondria β-oxidation of fatty acids for ATP production [37]. This could be the alterative survival mechanism as to why blockade of glucose uptake by cytochalasin B (see Fig 6B) did not significantly affect astrocytic cell death observed in figure 5B. We demonstrated that astrocytes showed more efficient adaptive oxidation of PV/PA than neurons for ATP production during glucose-deprived stress condition. We propose that METH targeted glucose transport function in neurons and astrocytes without severely affecting Krebs cycle, because oxidation of PV/PA was still active in these cells even after METH exposure in glucose deprivation. These findings suggest that pyruvate dehydrogenase that converts pyruvate to acetyl-coenzyme A was not the primary target of METH exposure.

Another interesting observation was that ALC prevented the METH-induced decrease in glucose uptake by stabilizing the GLUT1 and GLUT3 protein levels. This can be possible if METH exposure interferes GLUTs glycosylation and ALC can stabilize the glycosylation by donating acetyl group to glucosamine. Thus, acetylglucosamine can glycosylate GLUT1 or GLUT3 even in the presence of METH. This is because glucose uptake and transport is possible only when the GLUTs are enzymatically glycosylated by acetylglucosamine. As expected, cytochalasin B decreased the rate of glucose uptake without affecting glucose transporter protein levels. These findings showed that cytochalasin B inhibited glucose uptake and transport by modulating the active binding sites of GLUTs and not by degrading the actual GLUTs protein contents. However, METH altered glucose uptake and GLUTs protein levels. It would be interesting to know whether decrease in glucose uptake and GLUT protein levels in these cell types were related to the impairment of glucose metabolism in brain as indicated in METH abusers [21].

Finally, the question is whether chronic METH abuse causes hypoglycemia due to blockade of glucose transport across the blood-brain barrier or causes hyperglycemia as a result of passive diffusion through defective BBB? Similar to our recent findings in animal model, Fujioka et al. (1997) reported that METH abuse causes a hypoglycemia in human brain [38]. Thus, this deficient glucose level compounded with the inability of brain cells to handle the available glucose (as demonstrated here) may likely be a possible underlying mechanism for neuronal degeneration in METH abuse. Our data also suggest that brain cells (neurons in particular) might still be deprived of glucose-derived energy even in hyperglycemic brain because under METH exposure these cells are unable to utilize glucose efficiently due to impairment of uptake and transport mechanisms. This is because handling of glucose for energy generation is accomplished by active process only. Thus, destruction of active process of glucose uptake and utilization is likely to contribute for neurodegeneration in hypo/hyper-glycemic brain in METH abusers.
